# Vincristine-based nanoformulations: a preclinical and clinical studies overview

**DOI:** 10.1007/s13346-023-01389-6

**Published:** 2023-08-08

**Authors:** Rahul Shukla, Ajit Singh, Kamalinder K. Singh

**Affiliations:** 1 Department of Pharmaceutics, National Institute of Pharmaceutical Education and Research-Raebareli, Bijnor-Sisendi Road, Sarojini Nagar, Near CRPF Base Camp, U.P 226002 Lucknow, India; 2 School of Pharmacy and Biomedical Sciences, Faculty of Clinical and Biomedical Sciences, University of Central Lancashire, Preston, PR1 2HE UK

**Keywords:** Vincristine, Nanomedicine, Pharmacokinetics, Pharmacodynamics, Preclinical and clinical usage

## Abstract

Vincristine (VCR) is a chemotherapeutic agent obtained from natural alkaloid plant source *Catharanthus roseus.* VCR has been significantly useful in treatments of lung cancer, lymphocyte-based leukaemia, glioblastomas and acute myeloid leukaemia. VCR attaches to tubulin fibrils and prevents filament polymerization that permanently led to mitosis inhibition in cancer cells. Clinically, VCR is administered to patients in multidrug combination to reduce adverse drug effects and potential blockage of bone marrow inhibition due to prescribed monotherapy. However, VCR possesses low cancer tissue affinity and at higher dose often led to irreversible neurotoxicity. Conventional VCR injectables are successfully used in clinics, but lack of controlled release, non-specific biodistribution and consequent off-target side effects are still major challenges. Currently, nanotechnological drug delivery systems are being explored for improvement of VCR pharmacokinetic profile and tumour-specific targeting. Various nanomedicine formulations such as liposomes, lipid nanoparticles, and polymeric nanocarriers of VCR have been studied under various in vitro and in vivo models. In this review, we have summarised the chemotherapeutic role of VCR, evaluated the mechanism of action, pharmacokinetics and challenges associated with VCR delivery. Moreover, application of VCR in nanomedicine and effect on anticancer efficacy in preclinical and clinical setting are also being discussed.

**Graphical Abstract:**

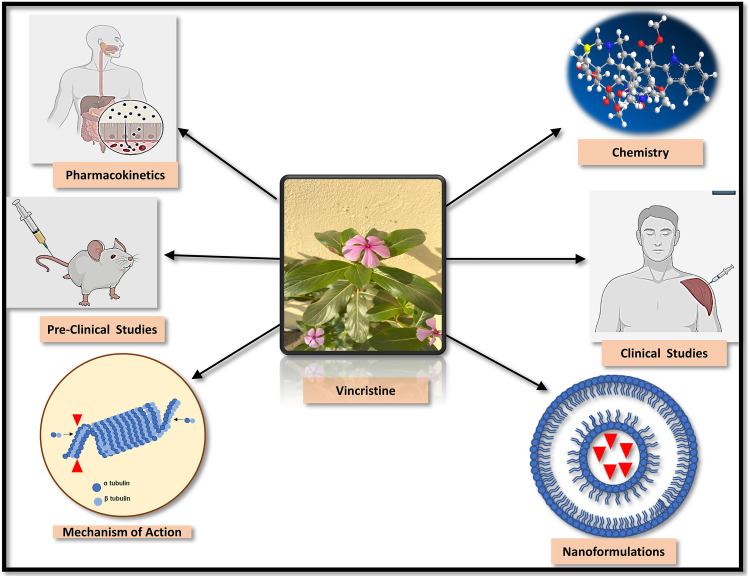

## Introduction

The vinca alkaloid vincristine (VCR) was first isolated from the medicinal herb found in Madagascar known as *Catharanthus roseus*. This cytotoxic drug was first discovered in 1985 and found useful in cancer therapy [1]. VCR is also referred to as leurocristine and promoted commercially as “Oncovin” [1, 2]. It is administered intravenously as chemotherapeutic agent for management of various type of melanomas including lung cancer, lymphocyte-based leukaemia, glioblastomas and acute myeloid leukaemia [3]. The VCR is mostly administered in combination with other chemotherapeutic agents. VCR combination regimens for Hodgkin cancer include Mustragen, Oncovin^®^, procarbazine, and prednisone called MOPP. Another combination regimen is cyclophosphamide, oncovin^®^, procarbazine, and prednisone referred to as COPP. Additionally, BEACOPP is an intensified combination therapy for Hodgkin cancer including bleomycin, etoposide, doxorubicin, cyclophosphamide, Oncovin^®^, procarbazine, prednisone. While for non-Hodgkin cancer, cyclophosphamide, doxorubicin, oncovin^®^, and prednisone (CHOP) is the preferred regimen [4]. Whereas Stanford dose regimen was introduced in patients affected with nephroblastoma, and acute lymphoblastic leukaemia. Although combinatorial therapy of VCR with prednisone, dexamethasone and L-asparaginase are used for the treatment of acute lymphoblastic leukaemia (ALL) and early-stage leukaemia [5]. In some cases, VCR is also used in the management of various haematological diseases including thrombocytopenic purpura and chronic idiopathic thrombocytopenia [6]. With the abovementioned advantages, VCR is associated with pharmacokinetic (PK) limitation such as low cancer tissue binding, rapid first pass metabolism by CYP3A4/5 enzyme, P-glycoprotein (P-gp) overexpression, non-specific biodistribution, short half-life and drug resistance [7, 8].

However, to overcome these challenges, currently nanotechnology is used to modify physical properties of a drug to achieve reduced side effects and target specific delivery. Ongoing research is focused to develop specialized targeting or active advancements that will increase effectiveness of anticancer medicines while lowering the adverse effects to normal tissues [9, 10]. These nanocarriers are prepared from non-toxic and safe excipients which aid in diagnosis and targeting of cancer cells [11]. Various lines of experiments have reported that nanoparticles (NPs) such as liposomes, polymeric NPs, metal-based NPs and lipid NPs are able to deliver the VCR to cancer cells. The clinical trials of VCR-loaded nanocarriers have also showed effective therapy in cancer patients. This review will summarize the chemotherapeutic role of VCR and evaluate its mechanism of action and pharmacokinetics. This article also discusses application of VCR in form of nanomedicine to improve its safety and anticancer efficacy in preclinical and clinical setting.

## Chemistry of vincristine

**Fig. 1:**
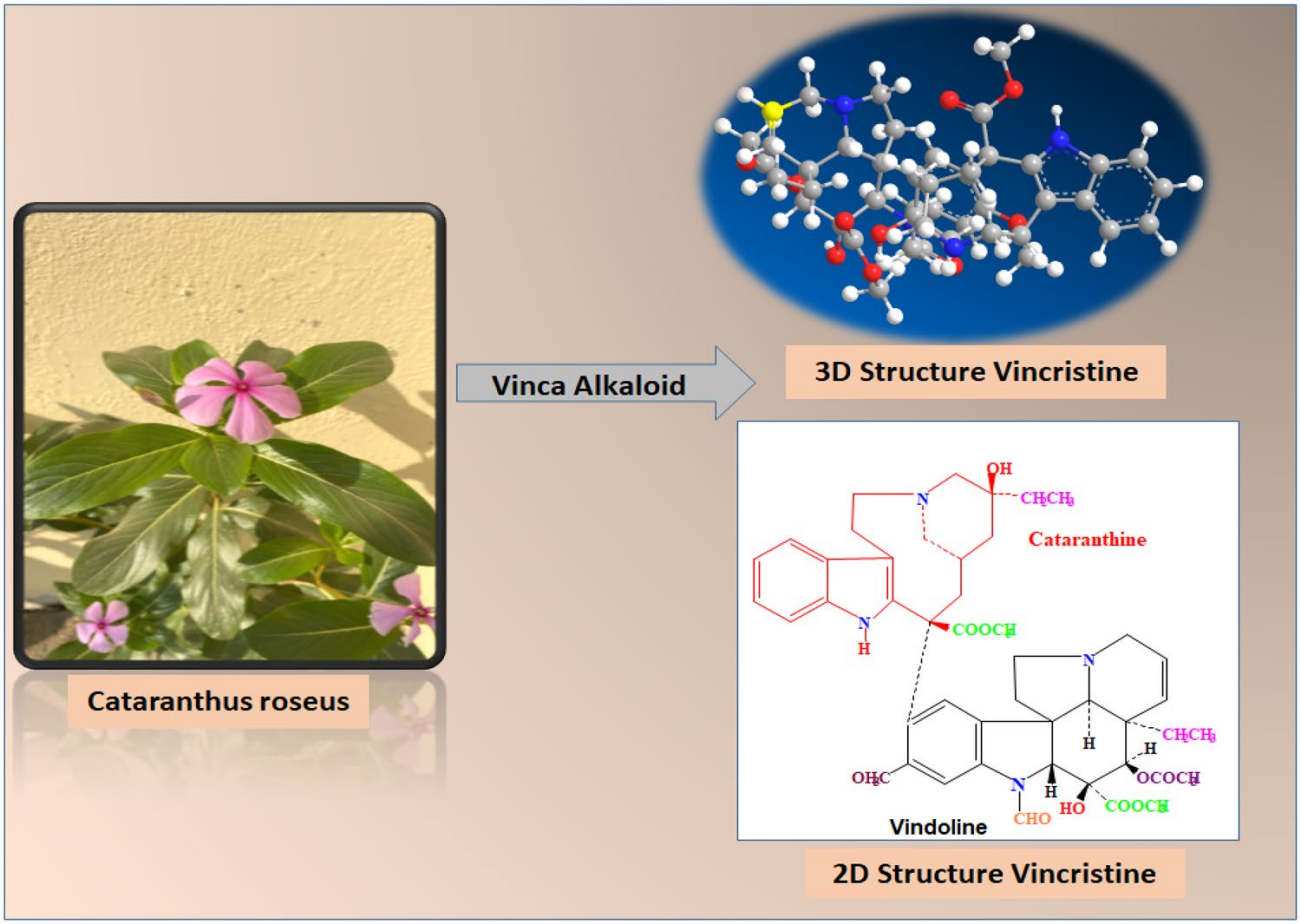
Natural source and chemical 2D and 3D structure of vincristine

VCR extracted from *Catharanthus roseous* (family *Apocynaceae*) which is a terpenoid indole alkaloid [12]. It is isolated via semi-synthetic coupling of indole alkaloids vindoline and catharanthine [13]. The molecular weight of VCR is 824.958 Da and its molecular formula is C_46_H_56_N_4_O_10_ [14]. VCR is synthesized using stereochemistry-driven mechanism for specific stereo selective at C_2_ and C_18_ in structure as shown in Fig. 1 [15]. The stereo selective carbons are essential for cytotoxic activity of VCR. To enhance the cytotoxicity, different functional groups are introduced in the structure. In vindoline structure acetyl group at C_4_ and hydroxyl group at C_2_ position is important. Loss of these groups leads to reduction in anticancer activity [16–18]. Similarly, hydrogenation or reduction at C_6-7_ position at VCR also diminishes oncolytic process in cancer cells. The vincristine has low aqueous solubility of 0.03 mg/mL, pKa of 10.85 and 3.36 log *p* value [19].

## VCR mechanism of action on cancer cells

**Fig. 2:**
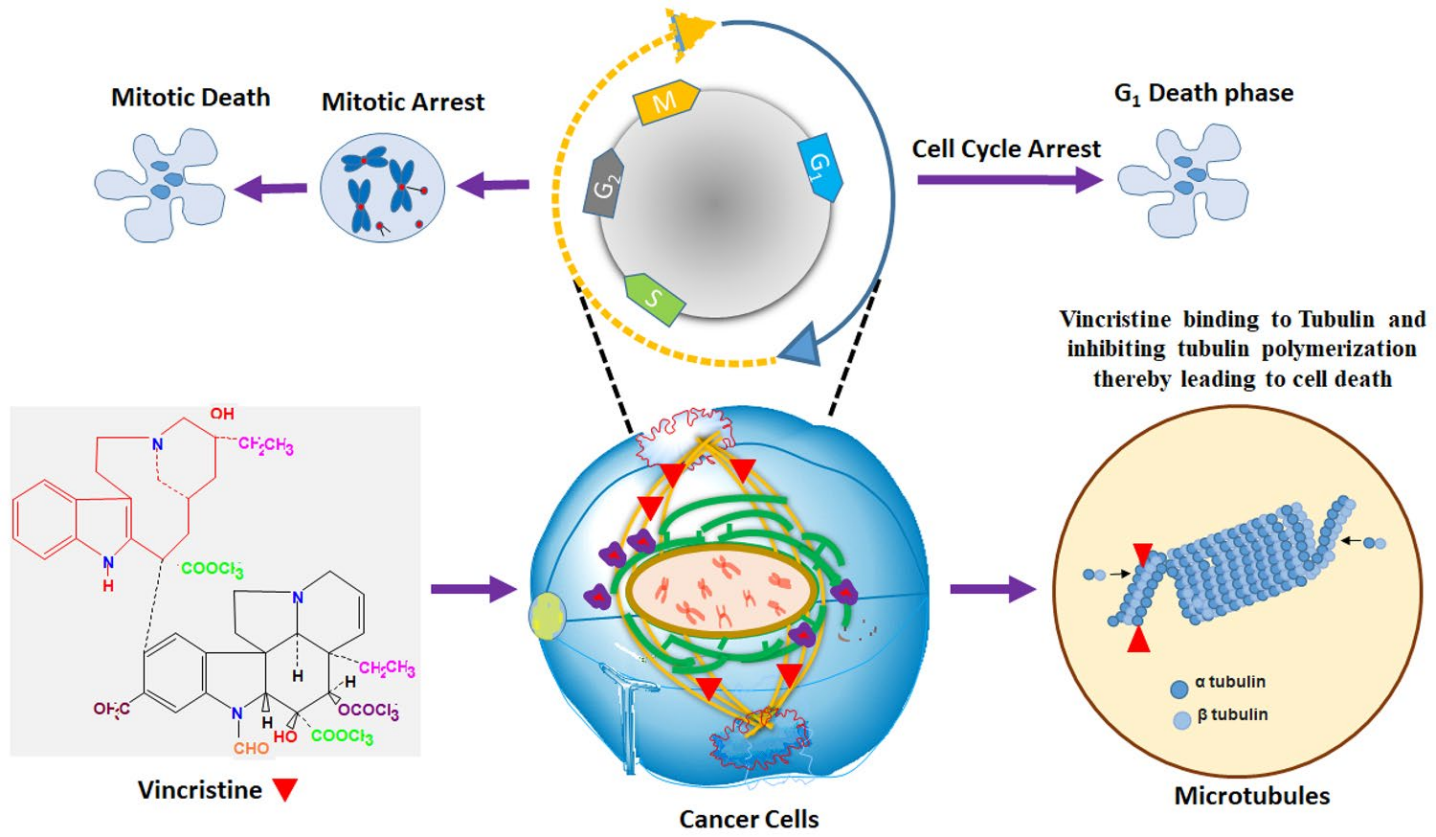
Vincristine mechanism of action on microtubule arrest for cancer cell cycle inhibition

The physiological pathway of VCR initiates by arresting metaphase of mitosis in cell cycle. In metaphase, polymerization of tubulin protein forms filament microtubules that enables cell detachment from chromosomes and enter the next phase of cell development. It mainly arrests polymerization of dimer tubulin protein which subsequently initiates apoptosis in cells as shown in Fig. 2. Along apoptosis, VCR also suppresses the growth and production of leukocytes from bone marrow [15, 20]. However, lower dose exposure or high clearance leads to reversible binding of VCR with tubulin protein. Therefore, the antitumor efficacy is dependent on dose and time of exposure [21]. It is also reported to have anti-angiogenesis and apoptosis induction activity in cancer cells [22]. VCR can induce the calcium ion ATPase activity, lipid and nucleic acid formation, and cell respiration [23]. Its hypoglycaemic effect is well studied and further reported in preclinical research [24]. The standard dose of vincristine is 1–1.4 mg/m^2^ every 3 weeks (intravenous bolus) [25]. At higher doses, VCR has shown a significant neurotoxic effect and inter-patient variability in few clinical interventions [26].

## Pharmacokinetics (PK) and metabolism of vincristine

**Fig. 3:**
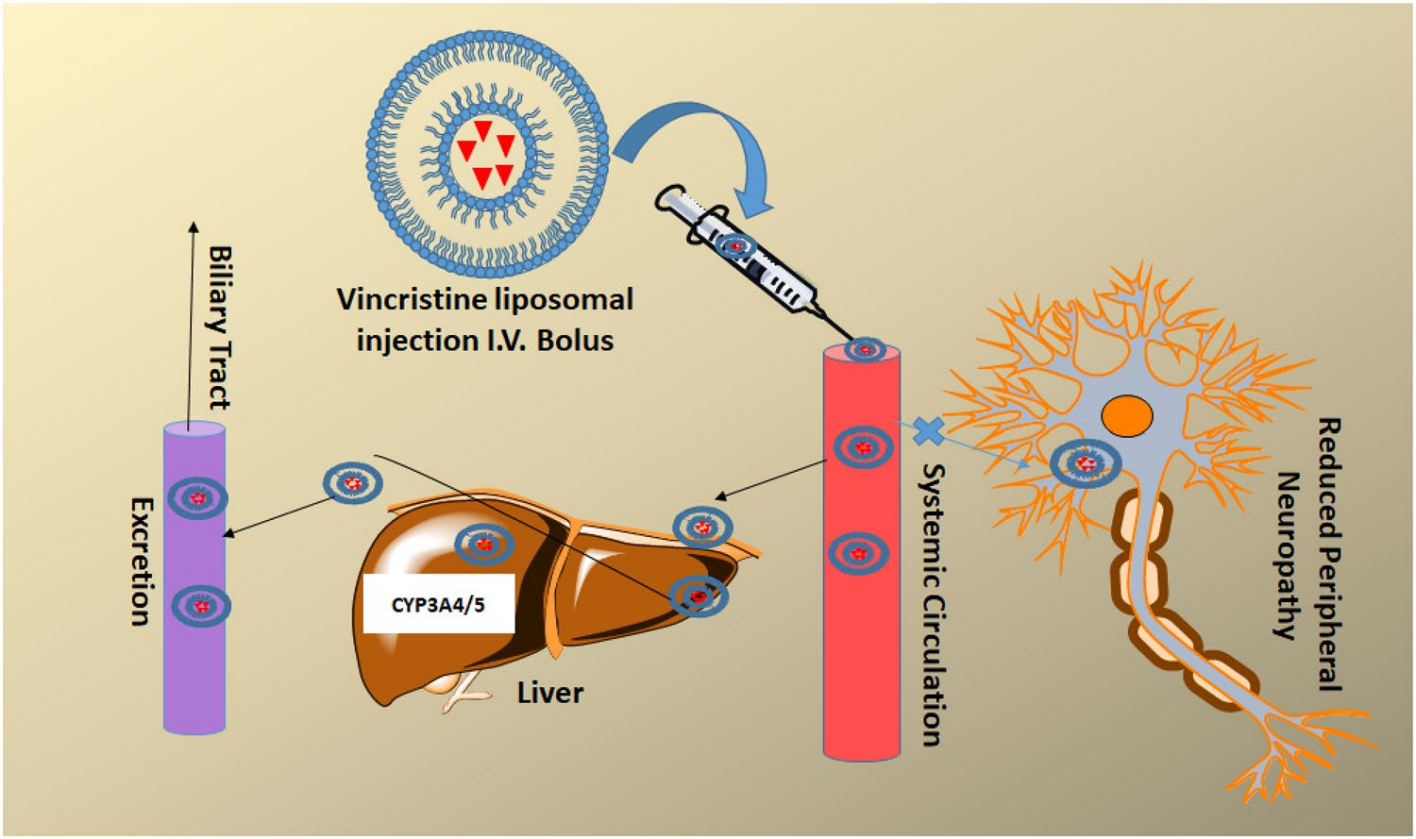
Following intravenous administration vincristine liposome passively diffuses through systemic circulation to liver and metabolized predominantly by CYP3A5 and excreted. Using VCR-liposomal formulation reduces the peripheral neuropathy

The single dose intravenous PK profile of VCR consists of drug concentration vs time graph has shown bi or tri-exponential kinetics with a very fast initial distribution followed by a longer elimination half-life. The clinical PK data of VCR showed long range parameters in human subjects such as half-life from 155 to 1500 min, volume of distribution (Vd) 57–420 L/m^2^ and clearance value is 82–482 ml/min/m^2^ [27–29] Hence, VCR has a large volume of distribution indicating a wide distribution in the body and extensive binding to tissues. PK of VCR revealed its affinity towards normal cells and less binding capacity with tumour tissues, which limits antitumour efficacy of the drug in vivo. It quickly absorbed by liver cells and reduces its delivery to cancer cells using a saturation process. Based on the kinetic data, hepatocytes uptake amount might be up to 500 times more than other tissues. Evidently, it accumulates heavily in the eye, and fatty tissues except the brain. Additionally, VCR has low affinity for the central nervous system compared to plasma [30–35]. These pharmacological properties limit clinical benefit of VCR by limiting plasma and cancer tissue drug exposure. VCR mostly metabolized through cytochrome CYP3A4 and CYP3A5 enzymes of liver shown in Fig. 3. Also, co-administration of another active ingredient increases or reduces the metabolism of VCR [36, 37]. The phenobarbital functions as CYP3A inducer which leads to rapid clearance of VCR from systemic circulation [38]. The clinical studies have shown that CYP3A5 exhibited an important role in P450 derived elimination of VCR and cytochrome genetic factors variability in patients [8, 39]. The high-pressure liquid chromatography analysis revealed that VCR was mostly excreted out from plasma to bile and urine [40].

## Challenges in clinical application of vincristine

Most common adverse effects associated with VCR therapy is peripheral neuritis, damage to lungs, high blood sodium levels, leucopoenia, alopecia, and headache. The major problem with VCR treatment is P-gp over expression and efflux of chemotherapeutics outside of cancer cells which leads to development of multidrug resistance. To tackle this issue, VCR formulated with P-gp inhibitor agent reduces the chances of drug resistance in cancer cells. To meet these essential problems, nano drug delivery system is used to enhance circulation time, improve aqueous solubility, optimise targeted delivery, and permit controlled release. The lung cancer cells have high VCR efflux rate than endocytosis rate and cells are more prone for development of multidrug resistance. Therefore, novel drug carrier system is explored to deliver the anticancer drugs to cancer cells or tissues using their small particle size and surface modification property. Bare or non-targeted NPs have ability to deliver compound through passive route which leads to non-specific bio-distribution of drugs. Pre-clinical research is progressing for development of target mediated drug delivery in cancer therapies as well as reducing toxicity to non-targeted healthy tissues [41–44]. These nanocarriers are used as formulation according to the physicochemical properties of drug [45, 46]. The food and drug administration for United States of America (USFDA) approved liposomal nanocarrier of VCR which was sold under brand name Marqibo^®^ [46]. The liposomal formulation was prescribed for adult patients with advanced, relapsed and refractory Philadelphia chromosome-negative (Ph-) acute lymphoblastic leukaemia (ALL) [19]. Further, pre-clinical studies for nanoformulation of VCR are summarised in Table 1.

**Table 1:** In vitro and in vivo studies on nanocarriers of vincristine

**Nano carrier and material**	**Objective and target**	**Drug**	**In vivo/in vitro model**	**Major outcome**	**References**
Liposomes DSPEG-Transferrin-	Liposomes DSPEG-transferrin used for co delivery of VCR and tetrandine for enhanced brain delivery	VCR and Tetrandine	Glioblastoma of Tumour Bearing Mice	Liposomes prolong the circulation time, obviously accumulate in brain tumour location, thus leading to a robust anticancer efficacy in glioma-bearing mice	[75]
Liposomes Egg sphingomyelin (ESM)/Choleste rol/N-palmitoylsphingosine-1{succinyl[metho xy(polyethylene glycol)2000] (PEG_2000_ceramide)	Liposomes is used to co-deliver VCR and quercetine to reduce JIMT-1 resistance in human breast cancer	VCR-Quercetin	The co-encapsulated liposome formulation demonstrated the most effective tumour growth inhibition in the JIMT-1 human breast tumour	The co-encapsulated liposome formulation exhibited significant antitumor activity at two-thirds of the maximum tolerated dose of vincristine, without significant body weight loss in the animals	[76]
Polymeric NPs PLGA-PEG-folic acid	The PLGA-PEG based nanocarrier grafted with folic acid used to target folic acid receptor on MCF-7 cell line. PEG acts as Pgp efflux inhibitor and increases VCR content in cell	VCR	MCF-7 cells and Pgp glycoprotein efflux inhibition activity	The therapeutic effects of the drug formulated in the NPs with surface modification could be 1.52 times, 3.91 times higher than that of PLGA–mPEG NPs and free vincristine sulfate, respectively	[61]
Polymeric NPs Chitosan-folic acid	Chitosan NPs are pH sensitive in nature and showed triggered release in pH 6.7 in tumour microenvironment	VCR	In vitro drug release study showed slow and sustained release of vincristine in phosphate buffered saline at pH 6.7	These vincristine loaded folic acid–chitosan conjugated nanoparticles could be used for targeted delivery against resistant cancer with some modifications	[43]
Polymeric NPs PLGA-Dextran Sulphate	Researchers have developed PLGA NPs coated with dextran sulphate to orally deliver VCR. The higher accumulation of VCR due P- glycoprotein inhibition due to dextran coating	VCR	Improved in vivo oral pharmacokinetics and enhanced VCR accumulation in MCF-7 cells	The new dextran might provide an effective strategy for oral delivery of VCR with improved encapsulation efficiency and oral bioavailability	[61]
Polymeric NPs PLGA	PLGA polymeric NPs are prepared to co-deliver VCR and verampil to inhibit Pgp glycoprotein in MCF-7 cells and reduce resistance	VCR and Verapamil	For multidrug resistance reversal is possible with VCR and verapamil found to important for maximum efficacy in MCF-7/ADR	Furthermore, the most effective tumour growth inhibition in the MCF-7/ADR human breast tumour xenograft was observed in the co-delivery nanoparticle formulation group in comparison with saline control, free vincristine, free vincristine/verapamil combinations and single-drug nanoparticle combinations	[42]
Polymeric NPs Accurin polymeric nanoparticle	Prostate specific membrane antigen (PMSA) is coated on polymeric NPs to deliver VCR and selective targeting of B-lymphocytes	VCR	Similar tolerability and anti-tumour activity data were obtained in MX-1 and Ramos xenografts. PSMA targeting of BIND-510 in C4-2 tumour xenografts resulted in a tumour growth inhibition (TGI) of (79%) compared to non-targeted VCR NPs which caused a 47% TGI at the same VCR dose level	BIND-510 as a targeted clinical therapy with the potential benefit of reduced toxicity and improved anti-tumour activity compared to currently available treatments in hematological as well as solid tumour settings	[59]
Solid lipid NPs (SLNs) of Stearic acid	Stearic acid based SLNs are used to deliver to anticancer agent VCR and temozolomide for enhanced anti-tumor efficacy in in vitro and in vivo model	VCR and temozolomide	VCR-SLNs and temozolomide NLCs were compared for their efficacy and found out higher tumour accumulation in both in vitro and in vivo model U87MG cells	VCR SLNs can deliver VCR and temozolomide into U87MG cells more efficiently, and inhibition efficacy is higher than VCR-SLNs	[81]
Hydrid lipid NPs Cetyl palmitate–Dextran sulphate	The hybrid lipid NPs are prepared using cetyl palmitate and dextran to encapsulate VCR. This nanocarrier is evaluated for pharmacokinetic and antitumor efficacy on animal and cell line model	VCR	SLNs enhanced the VCR sulphate cytotoxic activity on MDA-MB-231 cell lines compared to free VCR. The in vivo animal studies have revealed improved pharmacokinetics compared to VCR solution	Cetylpalmitate SLNs with dextran could produce high VCR-loaded SLNs suitable for delivery of anticancer drugs to brain tumours	[82]
Hybrid lipid polymer NPs PLGA, cholesterol, stearic acid, and PEG_2000_-DSPE	Hybrid lipid polymer NPs are prepared using PLGA stabilized by PEG_2000_-DSPE to deliver VCR and quercetin Non-Hodgkin’s lymphoma	VCR and quercetin	The nanocarriers showed higher antitumor efficacy Raji or Raji/VCR cells in combination	Co-encapsulation of VCR and QN in the same LPNs has potential as a novel therapeutic approach to overcome chemo-resistant lymphoma	[83]
Lipid NPs Distearoyl phosphatidyleth anolamine (DSPE)	DSPE based lipid NPs used to encapsulate Doxorubicin, Gemcitabine and VCR and evaluated deliver in lymph node based Raji cells for cytotoxicity	Doxorubicin, Gemcitabine and VCR	Raji cells were as in vitro and xenograft model. The combination of VCR, DOX and GEM increased the antitumor efficacy and bioavailability	The resulting Doxorubicin Gemcetabine VCR NLCs could be an efficient antilymph cancer agent and could be developed further for the treatment of other tumours	[84]
Inorganic NPs Chitosan-Silver	-The chitosan encapsulated silver NPs and VCR is used to evaluate activity on A459 and Madin-Darby canine kidney cells	VCR	A459 and Madin-Darby canine kidney cells	Significant cytotoxicity was observed in A549 cells than Madin-Darby canine kidney cells at *p* > 0.05 when incubated with VCR loaded Chit@AgNP, promoting high specificity towards cancer cells	[88]
Inorganic Nanocarrier Gold	VCR sulphate-conjugated gold NPs liposomes are used to prepare light based stimuli for enhanced antitumor efficacy	VCR	Showed higher cytotoxicity Hela cell in vitro and Tumour bearing animal model	Treatment with the prepared liposomes coupled with UV light exposure produced greater antitumor effects in nude mice and reduced side effects, as compared with free vincristine sulphate	[87]
Inorganic Nanocarrier Hydroxyapatite	VCR-loaded hydroxyapatite NPs used as potential delivery system for bone cancer therapy	VCR	The chorioallantoic assay revealed reduction in the angiogenesis of fertilized egg which is promising prospect for the nanocarrier in cancer therapy	Findings presented in this paper suggested that VCR-loaded hydroxyapatite has a promising future as a nanocarrier for bone cancer treatment	[89]

## Nanoformulation of vincristine

**Fig. 4:**
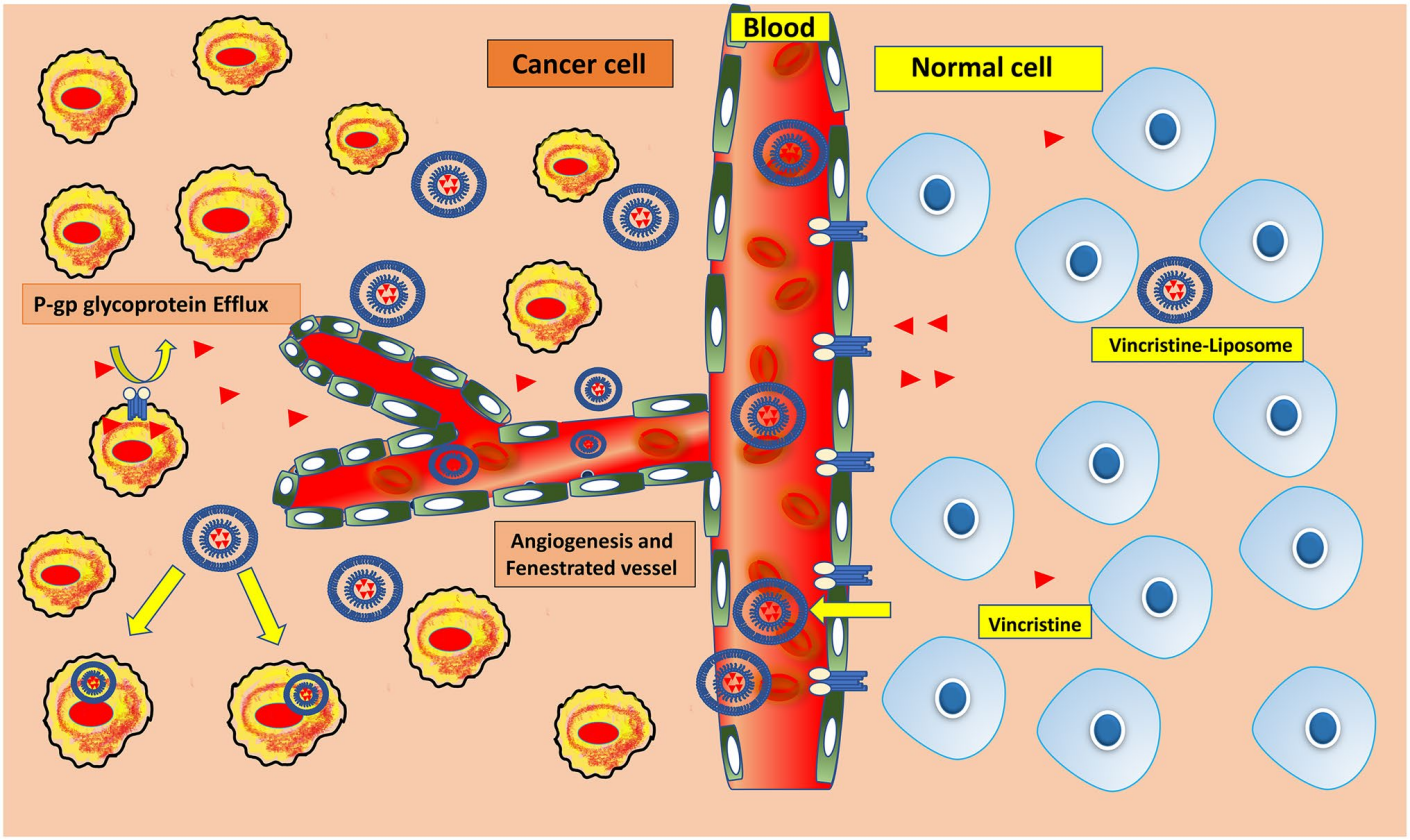
Enhanced permeation and retention (EPR) of VCR-liposomes in cancer cells compared to normal tissues. Vincristine liposomes extravasates into tumour and enhances the accumulation of drug at tumour site

**Fig. 5:**
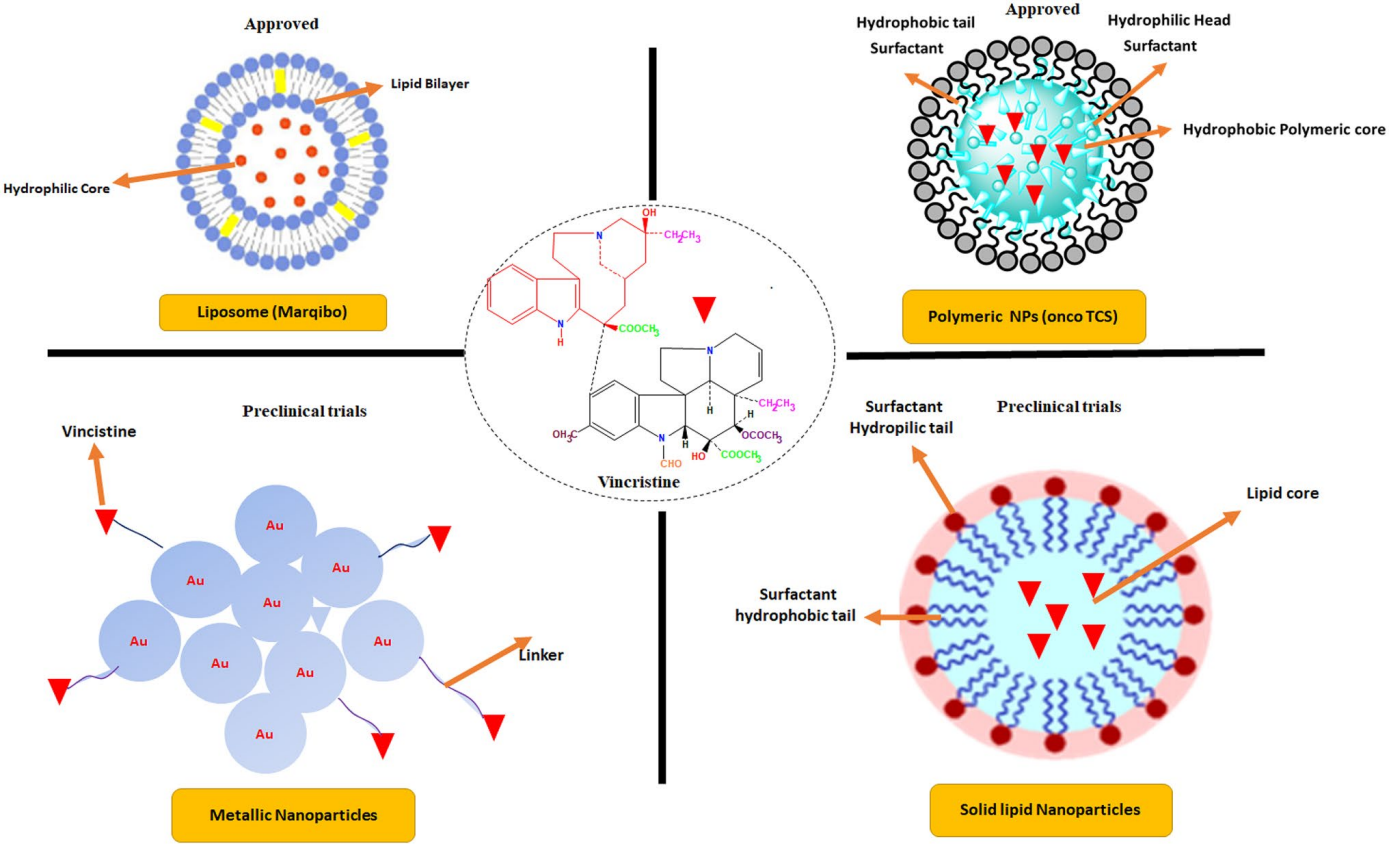
Types of vincristine nanocarriers used for evaluation on clinical and preclinical models

Nanomedicine has changed the features of drug delivery systems and transformed their potential in cancer therapy such as reduced side effect, target specific delivery and higher efficacy through enhanced permeation and retention (EPR) as shown in Fig. 4 [47, 48]. To acquire these goals, anticancer drug encapsulated nanocarriers attached with monoclonal antibodies or targeting ligand show enhanced anticancer activity in in vitro or in vivo models. Nanocarriers for drug delivery are mostly developed to decrease adverse drug reactions and site-specific targeting. Also, nanocarriers improve active pharmaceutical ingredient’s pharmacokinetic profile, reduces multidrug resistance and help in administration of drug through oral route. The nanocarriers consist of three dimensions in shape including external structure, peripheral and inner structure. Based on nanomaterials used for fabrication, nanocarriers have been developed including liposomes, polymeric NPs, metallic, and lipid NPs as shown in Fig. 5.

Numerous techniques have been used for the preparation of nanocarrier which depends upon the unique characteristics of material used for the formulation. Conventionally, there are four different generations of nanocarriers, in which the first generation NPs are bare nanocarriers (without surface modification) rapidly cleared from the systemic circulation [49–52]. In the second generation NP, the surface is conjugated with hydrophilic functional groups to form stealth barrier which reduces protein adsorption and prolongs circulation time. These second generation nanocarriers advanced into multifunctional tool including therapy, diagnosis and targeted delivery which are placed under the fourth generation. Whereas, third generation nanocarriers surfaces are modified with target specific ligand have affinity for certain receptors on cells or tissues [53–57].

## Types of vincristine nanoformulation

### Polymeric nanoparticles

Polymeric nanocarriers are prepared using biocompatible high molecular weight polymers to encapsulate biopharmaceutical class II or hydrophobic drugs. Most of the USFDA approved or marketed nanoformulation are liposomes and polymeric NPs. These polymeric NPs generally have particle size in the range of 100 to 500 nm for intended use as cancer targeting agents. Nanocarriers are designed according to factors such as stability, large scale productivity and nanoparticle-related toxicity. The large-scale production is important in progress of polymer nanoformulation. Thus, polymeric nanocarrier in clinical domain should be biocompatible and biodegradable in the human body [58]. In 2007, novel VCR polylactide polyethylene glycol (Accurin) based NPs were developed by BIND Biosciences. This nanocarrier is attached with prostate specific membrane targeting antigen (PSMA) for site-specific delivery of VCR. BND-510 enhanced drug concentration at prostate cancer site with controlled release and improved pharmacodynamics. Results showed significant tumour size reduction after treatment with BIND510 compared to free VCR injectable formulation in athymic mice implanted with breast cancer (MX-1), non-small cell lung cancer (NCI-H460), nasopharyngeal carcinoma (KB), PSMA-expressing prostate cancer (C4-2) and Burkitt’s lymphoma (Ramos) xenograft models. It also increased therapeutic efficacy and reduced adverse effects such as neuropathy. BIND Biosciences presented these findings in Nov 2015 at a conference on molecular targets and cancer therapeutics [59]. Till now, many VCR polymeric NPs are developed and studied with combination or single drug for cancer therapy. Wang et al. 2019 developed folic acid and R7 peptide anchored pegylated PLGA NPs for delivery of VCR sulphate to tumour site and overcome multidrug resistance. These polymeric nanocarriers enhanced the drug uptake in MCF-7 cells through folic acid receptor mediated endocytosis, whereas, R7 peptide conjugation in PLGA nanocarrier leads to higher accumulation of VCR in tumour due to Pgp efflux inhibition [60].

Similarly in another article, folic acid–conjugated chitosan NPs were prepared for encapsulation of VCR. This VCR loaded chitosan nanocarrier demonstrated particle size of 897.5 nm with polydispersity index of 0.738 and zeta potential of + 11.2 mV. The drug encapsulation was confirmed by Fourier transform infrared spectroscopy while crystalline structure of drug within the NPs was confirmed by X-ray diffraction. The conjugation ratio of folic acid to chitosan of 4:25 showed highest encapsulation efficiency and drug loading of VRC. These nanocarriers are found to be stable at 7.2 and 7.6 pH and at high temperatures [43].

In another study, Ling et al. found enhanced drug entrapment efficiency and plasma drug concentration of VCR using poly (lactic-co-glycolic acid) and Dextran sulphate graft polymer-based NPs (PLGA-DPNs). The nanocarrier of this polymer was prepared using nanoprecipitation method. In this conjugate, dextran sulphate imparts negative charge to nanocarrier lead to increase in encapsulation efficiency to 93.6% for VCR. The entrapment of VCR in Dextran-PLGA complex is based on electrostatic interaction. After oral treatment of VCR plain drug and VCR-PLGA-DPNs to mouse for in vivo bioavailability studies showed that the apparent AUC of nanocarriers was around 3.3 times higher than free drug. The intracellular uptake of nanocarriers determined using fluorophore tagged fluorescence microscopy imaging in P-glycoprotein (P-gp) over-expressing MCF-7/Adr breast carcinoma cells was 12.4-fold higher than that of free VCR revealing that P-gp mediated efflux of VCR was considerably reduced by the graft polymer–based NPs [61].

Multidrug-resistant melanomas can be cured through combinatorial approach having cytotoxic drug and chemosensitizer. To offer such prospect verapamil, P-gp efflux inhibitor and chemosensitizer were co-encapsulated with VCR in PLGA nanocarriers. This nanocarrier was prepared using O/W emulsion solvent injection technique. This resistance reversal using VCR and verapamil showed higher anticancer efficacy in MCF-7/ADR cells. Alone, VCR-PLGA and Verapamil-PLGA nanocarriers exhibited less therapeutic efficacy compared to combinatorial delivery. This approach also reduces adverse drug reactions and drug–drug interactions [42]. Chen et al. worked on folic acid conjugated PEG-PLGA nanocarrier encapsulated with VCR sulphate for treatment of breast cancer. The double emulsion solvent evaporation technique was employed to prepare these nanocarriers. Folic acid surface modification of nanocarrier enhanced the MCF-7 cell uptake and cytotoxicity in 24 h. Due to surface modification of nanocarrier VCR IC_50_ reduced from 3.91 to 1.5 µM as compared to non-targeted NPs in MCF7 cells [62].

To improve lymphatic tissue targeting, Tan et al. used VCR sulphate encapsulated in polybutyl-cynoacrylate (PBCA) nanocarriers prepared through emulsion polymerization method using pluronic-127 (PF-127) as stabilizer (PF-127-VCR-PBCA) and non-coated (VCR-PBCA) NPs. This nanocarrier was evaluated in in vitro and in vivo animal models for lymphatic route targeting. The finding of this study revealed that as compared to free VCR, both PF-127-VCR-PBCA and VCR-PBCA exhibited sustained release behaviour. The cell uptake studies on Raji lymphoblast cells showed higher internalization of PF-127-VCR-PBCA as compared to VCR-PBCA nanocarrier. Reduced clearance and higher bioavailability of VCR were observed in lymphatic circulation of animal group treated with PF127-VCR-PBCA-NPs. In nude mice with xenograft of human Burkitt’s lymphoma, PF127-VCR-PBCA-NPs surpassed VCR-PBCA-NPs or free VCR in antitumour efficacy study. According to these results, surface coated polymeric nanocarriers might be effective delivery systems for anticancer drugs used to treat malignant lymphoma and metastatic cancers [63].

### Lipid nanoparticles

- Liposomes
  Liposomes morphologically consist of bilayer vesicles and hydrophilic core composed of phospholipids (phosphatidylcholine or phosphatidylserine) and cholesterol. Liposomes lipidic bilayer encapsulates hydrophobic drugs and inner hydrophilic core is able to entrap water soluble moieties [64]. There are a number of injectable liposomal formulations currently used in clinics including Doxil (doxorubicin liposomes) and Onvidye (Irinotecan liposomes) which encapsulate anticancer drugs and antimicrobial amphotericin B sold under brand name (Ambisome manufactured by Astellas Pharma), and opioid pain killer morphine sulphate (Depodur sold by Pacira Pharma) [65, 66]. The major advantage with liposomal formulation is their safety along with translational applicability and possibility of large-scale production. Also, they exhibit higher storage stability at different environmental conditions with drug loading capacity for both hydrophilic and hydrophobic drugs. Liposomes are explored for their improved drug delivery to the targeted site and reduced dose related adverse effects [67, 68]. In case of VCR, encapsulated liposomal formulation reduces IC_50_ dose compared to other traditional delivery systems. Liposomes have merits to entrap drug inside the liposomal core, reduces systemic side effects and toxicity [69]. VCR sulphate liposomal formulation Marqibo exhibited lower adverse effects and higher bioavailability of VCR at the targeted tumour site. Marqibo^®^ is composed of cholesterol and egg sphingomyelin (60:40) weight ratio which facilitated drug loading of VCR sulphate and resulted in liposomes of 115 nm. Marqibo^®^ showed prolonged plasma circulation time with increased accumulation in tumour tissue by preferential extravasation from fenestrated vasculature due to EPR effect. This formulation decreased the chances of clinically reported haematological toxicity associated with VCR treatment. Even though there are no signs of haematological toxicity observed at double dose of this liposomal formulation. Marqibo^®^ was used in combination with myelosuppressive agents for patients affected with non-tolerated peripheral thrombocytopenia [70].
  For therapy of relapsed non-Hodgkin cancer, another VCR liposomal formulation is under study named Onco TCS (Inex Pharmaceuticals) [71]. This VCR liposome exhibited higher bioavailability and enhanced plasma resident time in systemic circulation. Onco TCS uses proprietary drug-delivery technology platform of Inex called the transmembrane carrier systems (TCS). It is a pegylated liposomal formulation which reduces the clearance of VCR and increases the half-life of the drug. It releases VCR from liposomal core in controlled fashion at tumour site for prolonged duration of time. These liposomes mostly accumulate in the tumour and lymph nodes rather than other normal tissues even at high doses which reduce the adverse drug reactions of the free drug. Patients treated with Onco TCS are found less affected with peripheral neuropathy as compared to conventional VCR formulation. In mice models bearing L-1210 and P-388 leukaemia’s are Onco TCS was found to be more effective than free VCR [46].
  The ligand anchored liposomal formulation mostly shows higher cytotoxic efficacy in in vitro C6 glioma cells comparison to free drug or conventional delivery system and non-targeted liposomal preparations. Iron transporting proteins in cells such as transferrin composed of plasma proteins which absorbs iron from systemic circulation is studied for tumour targeting. Transferrin (TF) entry through biological membranes for critical activities such as cellular maturation and overexpression of receptor are found in malignant cancer cells [72–74]. TF-modified liposomes are developed to target brain endothelium cells for glioblastomas treatment. During preparation of such liposomes, transferrin is appended on the surface of liposomes encapsulated with tetrandrine and VCR. Article reported by Song et al. has co-delivered tetrandrine and VCR using TF modified 1, 2-Distearoyl-sn-glycero-3-phosphoethanolamine-Poly (ethylene glycol) (DSPE-PEG)-2000 liposome for brain delivery and glioblastoma treatment. The outcomes exhibited by transferrin surface modified liposomes increased the delivery of both drugs and inhibited multidrug resistance, extended half-life, higher intracellular uptake, reduced malignancy, decreased non-specific bio-distribution observed in tumour-bearing mice. In this study, liposomes prolong the circulation time, obviously accumulate in brain tumour site, thus leading to a robust anticancer efficacy in glioma-bearing mice [75].
  In a study, VCR and quercetin were delivered using liposomes to study the synergistic cytotoxic activity and pharmacokinetic profile for effective therapy of breast cancer. In JLMT-1, tumour bearing animal model was used, the results were compared between liposomal formulation control, free quercetin, and free VCR-treated animals which showed less effective in tumour growth reduction. Efficacy studies showed enhanced chemotherapeutic activity at 66% of VCR tolerable dose, with negligible adverse drug reactions and no significant vigour loss. This co-encapsulated liposome formulation exhibited significant antitumour activity at two-thirds of the maximum tolerated dose of vincristine, without significant body weight loss in the animals [76].
  Webb et al. reported fabrication of a vincristine liposome nanocarrier with markedly improved physicochemical and pharmacokinetic features. Liposomal VCR nanocarrier based on cholesterol and sphingomyelin was compared with distearoylphosphatidylcholine-based liposomes. Both liposomes were evaluated in terms of in vitro and in vivo pharmacokinetic and pharmacodynamics studies. In vitro stability of sphingomyelin-cholesterol liposomes is substantially higher compared to distearoylphosphatidylcholine-based liposomes. In PK studies, sphingomyelin-based liposomes showed 25% availability of encapsulated VCR in systemic circulation as compared to 5% retention in distearoylphosphatidylcholine-based liposomes. The higher retention is related to enhanced accumulation of VCR in peritoneal ascetic murine P388 leukaemia tumours and in subcutaneous solid A431 epidermis squamous carcinoma human xenograft models [77].
  Webb et al. reported fabrication of a vincristine liposome nanocarrier with markedly improved physicochemical and pharmacokinetic features. Liposomal VCR nanocarrier based on cholesterol and sphingomyelin was compared with distearoylphosphatidylcholine-based liposomes. Both liposomes were evaluated in terms of in vitro and in vivo pharmacokinetic and pharmacodynamics studies. In vitro stability of sphingomyelin-cholesterol liposomes is substantially higher compared to distearoylphosphatidylcholine-based liposomes. In PK studies, sphingomyelin-based liposomes showed 25% availability of encapsulated VCR in systemic circulation as compared to 5% retention in distearoylphosphatidylcholine-based liposomes. The higher retention is related to enhanced accumulation of VCR in peritoneal ascetic murine P388 leukaemia tumours and in subcutaneous solid A431 epidermis squamous carcinoma human xenograft models [77].

(b) Lipid-based nanoparticles
  Lipophilic active pharmaceutical ingredients can be entrapped in lipid bilayer of liposomes as described previously; however, certain drugs are unable to maintain efficient drug release from liposomes. Lipid NPs have emerged as suitable nanocarriers comprising of lipid core stabilised with suitable surfactants. The lipid NPS are classified into two groups (a) solid lipid NPs (SLNs) and (b) nanostructured lipid carrier (NLCs) [78]. These nanocarriers are able to efficiently encapsulate hydrophobic drugs and maintain controlled release in plasma for more than 24 h. To prepare these nanocarriers, various techniques are used such as solvent injection, probe sonication, high shear homogenization and high-pressure homogenization. Examples of anticancer drugs encapsulated in NLCs are doxorubicin, topotecan, paclitaxel and SN38. Anticancer drug gemcitabine is grafted with carbonyl group to increase the lipophilicity and encapsulation in lipid based nanocarriers [79, 80]. In this study, temozolomide and VCR-loaded glyceryl behenate and Cremphor ELP NLCs are prepared through solvent diffusion method. This formulation is evaluated on U87MG xenograft glioma bearing mice for anticancer efficacy studies. VCR-NLCs and temozolomide NLCs are compared for their efficacy and found out higher tumour accumulation in both in vitro and in vivo model. The dual drugs-loaded NLCs showed higher efficacy than single drug-loaded NLCs. These VCR SLNs can deliver VCR and temozolomide into U87MG cells more efficiently, and inhibition efficacy is higher than VCR-SLNs [81].
  Aboutaleb et al. formulated SLNs of VCR using cetyl palmitate and dextran sulphate via microemulsion technique. These SLNs critical process parameters were optimized through Box Behnken design. SLNs are evaluated for their particles size, zeta potential, and morphology and crystal nature. The encapsulation efficiency increased to 93% and sustained release behaviour of VCR was observed in tumour microenvironment. SLNs enhanced the VCR sulphate cytotoxic activity on MDA-MB-231 cell lines and compared with free VCR-treated groups. The in vivo animal studies have revealed improved pharmacokinetics with longer circulation and prolonged residence time as compared to free VCR. Coumarin-6 encapsulated SLNs were used as probe for brain uptake imaging for nanocarriers revealing almost fivefold brain accumulation in comparison to free fluorescent probe. SLNs with dextran could produce high VCR-loaded SLNs suitable for delivery of anticancer drugs to brain tumours [82].
  In another report, lipid polymer hybrid nanocarriers were developed for co-delivery of VCR and quercetin for synergistic treatment of lymphoma. This lipid-polymer nanocarrier is made of DSPE-PEG and PLGA polymer exhibited nanometric size, high negative zeta potential and controlled in vitro drug release. Co-delivery of VCR and quercetin enhanced the antitumour efficacy and showed synergistic cytotoxicity. They found that lipid nanocarriers also aided in the treatment of chemo resistant lymphoma [83].
  Radiotherapy and chemotherapy are majorly preferred treatment by clinicians for treatment leukaemia of B-cells. Combinatorial approach of doxorubicin, gemcitabine and VCR is normally applicable in B-cell lymphoma therapy. Therefore, Shuqin et al. synthesized prodrug of doxorubicin-gemcitabine which is co-encapsulated with VCR in NLCs and evaluated on in vivo B-cell lymphoma model. The toxicity associated with these anticancer drugs was also evaluated after treatment. The prodrug and VCR encapsulated NLCs exhibited enhanced cytotoxicity in lymphatic B-cells bearing animal model in comparison to single chemotherapeutic agent. The delivery of three chemotherapeutics in single system enhanced anti-lymphatic cancer treatment and would be a useful future strategy for clinical studies [84].
  The glioblastoma multiforme is one of the most malignant types of brain cancer with poor prognosis. In preclinical studies, various nanocarriers including liposomes, solid lipid NPs and polymeric NPs have been studied for delivery of chemotherapeutic drugs for this difficult-to-treat brain tumour. Zhang et al. developed lactoferrin and arginine-glycine-aspartic bi-ligand modified NLCs for delivery of temozolomide and VCR for glioma therapy. The physical characteristics such as particle size, zeta potential and entrapment efficiency were evaluated. The drug release profile, cellular uptake, cytotoxicity, tissue distribution, and antitumour activity of NLCs are further investigated which showed higher drug accumulation and enhanced antitumour efficacy in vitro and in vivo models [85].

(c) Inorganic nanoparticles
  The inorganic nanocarriers are mostly used for their diagnostic applications and have properties including reactive oxygen species generation and superparamagnetic nature of iron NPs. They have characteristics to form chemical bond with fluorescent dyes and functions as efficient theranostic. The cytotoxic drugs attached to iron NPs accelerate absorption, clearance is delayed from circulatory system and the EPR effect due to passive targeting boosts tumour retention. The metallic NPs (Ca, Fe, Au, Ag, Zn, and Ti) can be manufactured in large quantities thus amenable for scale-up. However, their tolerance and lack of in vivo accumulation over time must be validated to ensure safety. Ferrous NPs are the most widely studied metallic NPs based on their magnetic qualities and imaging ability. Conjugation is a possible method for theranostic applications in cancer therapy combining properties of the therapeutic and diagnostic in single metallic NPs platform [86].
  In a study, gold-VCR complex NPs are encapsulated into the liposomes to increase the anticancer activity. The VCR is physically coated on the surface of gold NPs which is identified using ultraviolet–visible spectroscopy, differential scanning calorimetry and Fourier transform spectroscopy. The liposome had the particle size 113.4 nm with photo-responsive VCR release properties into in vitro systems. This VCR-gold liposome cell uptake and distribution in Hela cells was observed using electron microscopy or confocal laser scanning microscope. The antitumour efficacy was evaluated on in vitro Hela cells and xenograft model of mice. Treatment with the prepared liposomes coupled with UV light exposure produced greater antitumour effects in nude mice and reduced side effects, as compared with free VCR sulphate [87].
  In another study, chitosan-encapsulated silver NPs having hydrodynamic size of 12 nm were used to deliver VCR to tumour cells. The nanocarrier showed triggered release under tumour microenvironment condition and able to deliver VCR in A459 cells efficiently. The drug loading of VCR in Chit@AgNPs was reported around 48% and zeta potential of − 11.7 mV. It exhibited a significant cytotoxicity in A459 and Madin-Darby canine kidney cells in comparison to plain VCR solution [88].
  Hydroxyapatite-based nanocarriers are non-immunogenic and biocompatible which accumulate in bone cells. Luisa et al. prepared a mesoporous VCR-loaded hydroxyapatite NPs (VCR-HANP) with 285 nm particle size and surface area of 103 m^2^/g. The amount of VCR entrapped in nanocarrier was quantified using UV–Visible spectroscopy demonstrate entrapment efficiency. The chorio-allantoic assay revealed reduced angiogenesis with VCR-HANP in fertilized egg indicating promising prospect for these nanocarrier in cancer therapy for bone cancer [89].

(d) Vincristine nanoformulation clinical perspective
  The clinical evaluation of anticancer drugs in the form of nanomedicine has drastically advanced in the current scenario. The evaluation of nanomedicines is not limited to only in vitro and in vivo animal studies, instead there are more than 1000 studies under clinical review [90]. Only for the liposomal drug formulation number of studies completed or actively taking place registered in database of the National Institute of Health including lurtotecan, docetaxel, VCR, cisplatin, irinotecan, camptothecin and other molecules are under new drug development program and currently under clinical trials [91]. Therapy for multiple myeloma includes VCR in combination with doxorubicin and dexamethasone (bolus or daily infusion). Additionally, it is recommended in paediatric oncology. VCR is more sensitive to neonatal malignancies, and pharmacotherapy doses in children are well tolerated [92].
  Faderl et al. proposed novel multidrug therapy of doxorubicin/cyclophosphamide/dexamethasone/VCR. Overall, 90 patients were enrolled between the age lines of 14–70 years with an average age of 34 years. The therapy regimen mostly consists of tolerated dose of VCR, dexamethasone, L-asparaginase and Pegylated asparaginase for 62 patients. Patients affected with around 78% patients were going through primary therapy. The initial recovery observed in 12.6 months’ time period under 78 month’s therapy. The number of individuals related with preliminary refractory disorder are 10% in number and 47% of patients recovered entirely between 18 and 80 days with an average of 29 days. In 1 month, around 8% of patients died with average of complete remission of 5 days. Further study showed 6% progression free survival in a 6-month therapy. The average complete remission for whole survival progression studies was 10.2 months. Patient progression rate of those with stem cell transfusion is recorded around 32%. The problems brought on by myelosuppression remained constant. Laparoscopy gastrostomy (PEG)-asparaginase was more efficient than L-asparaginase administered patients [93].
  In another study, overall 54 individual patients having metastatic melanoma enrolled by Deitcher et al. (2014) to evaluate the toxicity in blood samples related with VCR sulphate liposome injection (VSLI) shown in Table 2. VSLI exhibited no relation with enhanced cell count for patients administered with 2.25 mg/mL dose at every 14^th^ or 7^th^ day. This liposomal formulation can be used in combination and multidrug therapy for patients of hemocytopenia. VSLI have ability to maximize and enhance the VCR concentration in vivo which increases the antitumour efficacy. Similarly, in 2nd group, 54 patients were treated with similar 2.25 mg/mL dose of VLSI every 2 weeks. In relation to group 2 average dose of VSLI was less for group 1. Also, total neutrophil count reduced from baseline value and increment in platelet count (group I 8.7 weeks vs group II 5.7 weeks). The study concluded that VSLI prescribed dose regimen reduces the chance of significant haematological adverse effects. The researchers reported that VSLI reduces the prevalence of clinically severe cytogenetic adverse events when used at the recommended dose. Almost doubling of the median dosage density had no appreciable impact on the frequency and seriousness of hematologic adverse events. When combined with antineoplastic drugs, VSLI may be appropriate for usage in patients who cannot tolerate peripheral blood cytopenia [64].

**Table 2:** Vincristine nanocarriers which are clinically approved

**Brand Name**	**Onco TCS**	**Marqibo** ^®^
Marketing Company	INEX Pharmaceuticals	Talon Pharmaceuticals
Type of Nanocarrier	VCR liposomal injection formulated with trans membrane carrier system technology	Sphingomyelin/cholesterol liposomes, with an approximate liposome mean diameter of 100 nm
Dose	2.0 mg/m^2^	2.25 mg/m^2^
Used in treatment	Relapsed Non-Hodgkin lymphoma and acute lymphoblastic leukaemia	Acute Lymphoblastic Leukaemia
Route of Administration	Intravenous	Intravenously over 1 h once every 7 days
Advantages	Patients treated with Onco TCS were found less effected with peripheral neuropathy	Used in combination with myelosuppressive agents for patients effected with non-tolerated peripheral blood cytopenia
References	[64]	[65]

Liposomal VCR formulation (Onco-TCS) found to be less neurotoxic when given in full dosages and extended half-life, reaching higher concentrations in tumour site and lymph nodes than in neurons. Around 51 clinical patients that have been reported in which 35 were evaluated for study. There were 21 men and the median age was 62 years (the range was 19 to 86). The study showed 11 patients were identified with motor or sensory neuropathy of grade 3–4, which led to the cessation of treatment for five patients. These five patients had a history of neurotoxicity, and two of the five had been previously treated with paclitaxel, platinum, or both. Three individuals had fevers without lethal fatalities. Liposomal VCR is effective and well tolerated in this cohort of patients with relapsed non-Hodgkin lymphoma. But patients which already received several prior treatments are associated with the chance of having neurotoxicity. These findings are verified, liposomal VCR may also be used with combination for therapy of Non-Hodgkin lymphoma patients which have not received any prior treatment [46].

## Regulatory perspective

As nanomaterials can have chemical, physical, and biological properties that differ from those of their larger counterparts, the US FDA shares with the public an overview of current FDA activities in regulating nanomaterials and nanotechnology-enabled products and guidance documents on its internet website [94]. FDA has issued a guidance document on the development of human drug products, including those that are biological products, in which a nanomaterial is present in the finished dosage form [95]. Nanotechnology may be used to create drug products in which nanomaterials serve a variety of functions, as active ingredients or inactive ingredients, including carriers loaded with an active ingredient. The inclusion of such materials may result in product attributes that differ from those of products that do not contain such materials, and thus may merit particular examination.

VCR conventional formulation, Oncovin is approved for indications of acute lymphocytic leukaemia, lymphoid blast crisis of chronic myeloid leukaemia, and Hodgkin and Non-Hodgkin lymphoma. VCR is suggested to be administered intravenously as a short 5 to 10-min infusion. Administration via any other routes may be fatal.

Liposomal formulation of VCR, Marqibo was approved by regulatory authorities US Food and Drug Administration (FDA) or the European Medicines Agency (EMA). Marqibo which is liposomal vincristine encapsulated in sphingomyelin and cholesterol at a molar ratio of approximately 60:40 (mol:mol) received accelerated FDA approval in 2012 for use in adults with advanced, relapsed and refractory Philadelphia chromosome-negative ALL or whose disease has progressed following two or more anti-leukaemia therapies. The accelerated approval of Marqibo for Ph-ALL required post-marketing clinical trial intended to verify the clinical benefit of Marqibo. However, post-marketing trial required to verify clinical benefit has not been completed, due to patient recruitment to fulfil the PMR appeared to be significantly challenging due to the treatment options that are currently available. Acrotech, the company who had marketing authorisation for Marqibo voluntarily requested that FDA withdraw approval of this application. On Acrotech’s request, approval of NDA 202497 for Marqibo (5 mg/5 mL), and all amendments are withdrawn as of May 2022 [96].

## Future perspective VCR nanofomulation

Based on clinical studies, VCR nanocarriers present huge opportunities as important therapeutic in treatment of various type of cancers. Furthermore, nanocarriers have revealed applications in in vivo system. Nanocarriers offers organ-specific drug delivery that ultimately increase range of theranostic use in future. The nanotechnology mediated cancer therapy results in tumour targeted delivery reducing non-specific distribution of chemotherapeutics and would potentiate and reverse acquired multidrug resistance. Targeted nanocarriers will be used to deliver VCR with immunostimulants in cancer therapy in course to target the main cancer cell communication channels, prevent immune evasion, and counteract chemo resistance mechanisms. For example, it has been reported that nano-encapsulated curcumin can be used in conjunction with VCR as a chemo sensitizing agent [97].

In other perspective currently the cutting-edge nanotechnology will be used to treat the vector borne cancer ailment. According to published report, the virus hepatitis B/C and human papilloma virus contribute nearly 12% incidence rate in tumour formation. Therefore, VCR nanocarriers will play a major role in the treatment of virus-related tumours in the future [89]. The decades of immunological studies and relation with nanocarriers revealed their effects and application in immune-cancer therapy and this knowledge will aid in reduction of cancer patient mortality and suffering. Despite the necessity for extensive study, finding the biomarkers for identifying individuals who would benefit from chemoimmunotherapy is crucial. Additionally, the mechanism of immuno-chemo therapy against supportive tumour stroma is unclear, particularly in individuals with advanced disease. Therefore, creating potent VCR nanocarriers is crucial to battling the illness. With the development of targeted delivery medications with precise RNA-based gene sequence complexes and theranostics treatment of cancer will be easier in the future. The target specific nanocarriers could also be combined with extremely intricate organelle molecular imaging compounds to advance examination and diagnostics in early cancer encounters, particulate tracking instantly, as well as imaging and monitoring the course of therapy [98].

## Conclusion

In summary, the major studies and technologies acquired by formulation scientists are used for development of VCR encumbered drug delivery system. Whereas, physiochemical, pharmacokinetic and toxicity-related challenges are important aspects to tackle prior to clinical use of VCR. The well-established nanotechnology is employed to overcome the limitation including adverse drug reactions, multidrug resistance and short half-life. In preclinical settings, various nanocarriers such as polymeric NPs, liposomes, lipid-based NPs and inorganic nanocarriers have been studied, that showed enhanced antitumour efficacy as compared to VCR solution. Using nanocarrier VCR might be co-delivered with immunosuppressive agents for patients affected with peripheral blood cytopenia. Therefore, the nanoformulation-related findings revealed that targeted and combinatorial drug therapy can significantly improve anticancer efficacy. In case of clinical approach of VCR nanoformulation, knowledge of their pharmacokinetics, metabolism and co-administration with other anticancer agents such as temozolomide, asparagine and dexamethasone will reduce emergence of multidrug resistance. Therefore, assessing the potential application of NPs has great promise for future research in anticancer therapy.
